# MicroRNA Expression Data Reveals a Signature of Kidney Damage following Ischemia Reperfusion Injury

**DOI:** 10.1371/journal.pone.0023011

**Published:** 2011-08-15

**Authors:** Michael D. Shapiro, Jessamyn Bagley, Jeff Latz, Jonathan G. Godwin, Xupeng Ge, Stefan G. Tullius, John Iacomini

**Affiliations:** 1 Department of Pathology, Sackler School of Graduate Biomedical Sciences, Boston, Massachusetts, United States of America; 2 Translational Immunology Science Center, Molecular Cardiology Research Center, Tufts Medical Center, Boston, Massachusetts, United States of America; 3 Department of Medicine, Brigham and Women's Hospital and Children's Hospital Boston, Boston, Massachusetts, United States of America; 4 Division of Transplant Surgery and Transplant Surgery Research Laboratory, Harvard Medical School and Brigham and Women's Hospital, Boston, Massachusetts, United States of America; 5 Program in Immunology and Department of Pathology, Sackler School of Graduate Biomedical Sciences, Tufts Medical Center and Tufts University School of Medicine, Boston, Massachusetts, United States of America; University of Colorado Denver, United States of America

## Abstract

Ischemia reperfusion injury (IRI) is a leading cause of acute kidney injury, a common problem worldwide associated with significant morbidity and mortality. We have recently examined the role of microRNAs (miRs) in renal IRI using expression profiling. Here we conducted mathematical analyses to determine if differential expression of miRs can be used to define a biomarker of renal IRI. Principal component analysis (PCA) was combined with spherical geometry to determine whether samples that underwent renal injury as a result of IRI can be distinguished from controls based on alterations in miR expression using our data set consisting of time series measuring 571 miRs. Using PCA, we examined whether changes in miR expression in the kidney following IRI have a distinct direction when compared to controls based on the trajectory of the first three principal components (PCs) for our time series. We then used Monte Carlo methods and spherical geometry to assess the statistical significance of these directions. We hypothesized that if IRI and control samples exhibit distinct directions, then miR expression can be used as a biomarker of injury. Our data reveal that the pattern of miR expression in the kidney following IRI has a distinct direction based on the trajectory of the first three PCs and can be distinguished from changes observed in sham controls. Analyses of samples from immunodeficient mice indicated that the changes in miR expression observed following IRI were lymphocyte independent, and therefore represent a kidney intrinsic response to injury. Together, these data strongly support the notion that IRI results in distinct changes in miR expression that can be used as a biomarker of injury.

## Introduction

Ischemia reperfusion injury (IRI) is a leading cause of acute kidney injury [1], [2], [3], that results in tubulointerstitial inflammation, cell death and a poorly understood repair process [4], [5], [6]. Renal IRI also leads to activation of innate and adaptive immune responses, resulting in tissue damage [7], [8]. The pathophysiology of injury and subsequent repair resulting from IRI has been extensively investigated, yet it is unknown how these processes are regulated and treatment is limited to supportive measures [2], [9].

microRNAs (miRs) are a class of small, noncoding RNAs that regulate gene expression [10], [11], [12], [13]. Given the emerging role of miRs in the control of various physiological processes, we hypothesized that miRs might play a critical role in the regulation of responses to renal IRI. To test this hypothesis we performed miR expression profiling on RNA isolated from the kidneys of mice that underwent unilateral warm ischemia and sham controls [14]. We determined that IRI leads to lymphocyte independent alterations in miR expression profiles, leading us to hypothesize that changes in miR expression could be used as a biomarker of renal injury resulting from ischemia and subsequent reperfusion. Here, we performed a detailed mathematical analysis of miR expression data using principal component analysis (PCA) in order to test the hypothesis that differential expression of miRs might serve as a biomarker of injury. We used spherical geometry to determine whether differences observed in miR expression between groups are significant.

## Results

### PCA of miR expression following unilateral warm IRI

To examine the possibility that changes in miR expression could be used as a biomarker for IRI, we observed changes in miR expression in C57BL/6 on days 1, 3, 5, 7, 14, 21 and 30 after warm ischemia (IRI) or sham surgery [14]. miR expression in naïve kidneys was used as a day 0 data point. We recorded expression of 571 miRs in miRBase 10.0. miRs with mean signal intensities under 200 were eliminated, leaving 144 miRs for analysis. Thus, we had a time series for each mouse group composed of points with 144 dimensions, i.e., points in **R** ^144^. We then analyzed these time series using principal component analysis (PCA).

**Figure 1:**
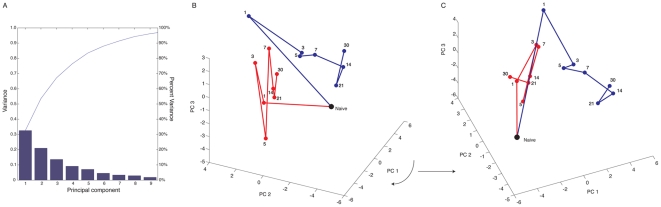
Principal component analysis of miR expression following unilateral warm IRI. Panel A, shown are variance plots of the first 9 PCs. The first 9 PCs account for over 90% of variance, while the first 3 PCs account for over 65% of variance. Panel B, three-dimensional plot of the first three PCs for mice undergoing either a sham procedure (red line), or IRI (blue line). Samples for naïve C57BL/6 mice are shown as a black dot. Numbers shown represent the time point analyzed in days. Panel C, shown is the plot from Panel B rotated clockwise in order to highlight differences observed for PC1 between samples. Because of the obvious constraints in depicting three-dimensional data as a two-dimensional figure we strongly encourage viewing original plots provided as .avi movies in the Supplemental Materials (Movie S1).

PCA revealed that greater than 95% of the variance observed between samples from C57BL/6 mice that underwent IRI and sham controls could be explained by the first 9 principal components (PCs), with the first 3 accounting for over 65% of variance (Fig. 1A). Thus, the majority of the variance observed in this data set can be captured in three dimensions. We analyzed the first three PCs in order to determine whether changes in miR expression following IRI could be distinguished from those observed in sham controls by generating three-dimensional variance plots. Three-dimensional variance plots revealed that relative to naïve controls, IRI and sham samples showed similar changes within the first 24 hours resulting in similar linear trajectories (Fig. 1B–C). After day 1, sham controls exhibited variance that appeared to fluctuate around the values shown for day 1. Presumably, this reflects alterations resulting from the effects of surgery itself. In contrast, the IRI samples exhibited changes in variance after day 1 that resulted in a visually distinct trajectory from that of sham controls (Fig. 1B–C), suggesting that miR expression following IRI is distinct.

### Defining the significance of distinct patterns of miR expression based on PCA

**Figure 2:**
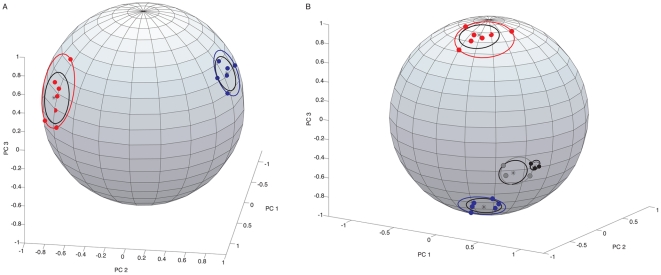
Determining the significance of distinct patterns of miR expression based on PCA. Panel A, shown are points on the surface of a sphere generated by, in the case of sham samples, placing the coordinates obtained for PC1-3 from naïve C57BL/6 kidneys at the center of a sphere and then projecting individual lines from the center of the sphere through each time point examined (days 1, 3, 5, 7, 14, 21 and 30) to the point where they crossed the surface of the sphere. The resulting seven points are shown in red for sham samples. The red circle shown is the smallest circle that contains all seven points, while the black inner circle represents the spherical standard deviation. For IRI samples we placed the coordinates of PC1-3 obtained for day 1 at the center of the sphere and then projected individual lines from the center of the sphere through each time point examined (days 3, 5, 7, 14, 21 and 30). The resulting six points are shown in blue. The blue circle shown is the smallest circle that contains all six points, while the black inner circle represents the spherical standard deviation. Panel B, analysis of the first three PCs of all IRI series as vectors emanating from the center of a sphere. C57BL/6 samples that underwent IRI are represented in blue. Rag-1^−/−^ and Rag-2/cγ^−/−^ samples that underwent IRI are shown as grey and black dots respectively. C57BL/6 sham control shown in red. Solid circles represent the smallest possible circle enclosing each group. Black lines represent standard deviation.

We next sought to assign a *P*-value to our visual assessment of the trajectories that our PCA produced in order to determine statistical significance. Each data point in the above PCA is a three-dimensional representation of the changes in miR expression for a given sample at a given time point. In particular, we sought to determine if there are well-defined sham and IRI directions. In the case of samples from mice undergoing a sham procedure, we considered the naïve control the center of a sphere, and projected lines from the center of the sphere through each data point (days 1, 3, 5, 7, 14, 21 and 30). We then intersected the lines with the surface of the sphere to generate a new set of points, and measured the radius of the smallest circle that contained all of them. (Fig. 2A). In the case of sham samples, the points generated lie within a circle of radius 21.7°. To determine whether the circle generated represents a direction, we took as our null hypothesis that the trajectory in question is a random walk. Using Monte Carlo methods we found that 600 in 10,000 random trajectories lie within a circle of equal or smaller size giving a *P*-value of 0.06. Thus, there does not appear to be a sham direction based on changes in the first three PCs.

For mice undergoing IRI, we considered the day 1 samples the center of the sphere, and then projected lines from the center of the sphere through the subsequent data points (days 3, 5, 7, 14, 21 and 30). We then intersected these lines with the surface of the sphere to generate a new set of points, and measured the radius of the smallest circle that contained all of them (Fig. 2A). We projected from day 1 because based on histological data and PCA it is apparent that both tissue damage and alterations in miR expression occur after day 1. For IRI samples, the points generated fall within a circle of radius 14.6° giving a *P*-value of 0.0069 based on the Monte Carlo methods used above. The question arises, are these two groups distinct? To test this, we compared the spread of each group on the sphere (as measured by their spherical standard deviation) to the angular separation between the two groups. Dividing the angular separation by the maximum of the spherical standard deviations gives a discrimination of 8.06. (For details of this computation see Supplemental Information.) Assessing this discrimination by Monte Carlo methods shows that the sham and IRI directions are distinct with a *P*-value of 0.005.

To validate this analysis, we performed similar calculations using all 144 reliably detected miRs rather than restricting our analysis to the first three PCs. We found that the radius angle of changes in miR expression in sham controls was 29.4° with a spherical standard deviation of 25.5°. Using Monte Carlo methods we obtained a *P*-value of 0.0001. Thus, analysis of the full 144 member set indicated that sham treatment results in a trajectory of variation in miR expression with a well-defined direction. Similarly, the radius angle of changes in miR expression in IRI samples was 23.8° with a spherical standard deviation of 22.3°. This resulted in a *P*-value of <0.0001, confirming that changes in miR expression over time following IRI represent a well-defined direction. The sham and IRI samples are separated by a radius angle of 107.3°, with a discrimination of 4.2. This corresponds to a *P*-value of <0.0001. Thus, the difference in directionality of sham and IRI data is not a random event, but rather reflects distinct alterations in miR expression. The results indicate that the pattern of changes in miR expression is observed as early as day 3 after injury and is maintained throughout the course of the experiment. This suggests that the overall changes in miR expression we observed are due to a single process of response to injury. We therefore conclude that miR expression may be useful as a biomarker of IRI.

### The injury signature defined by PCA of miR expression data reflects a lymphocyte independent process

To examine the extent to which lymphocytes may affect miR expression profiles, we performed sham and IRI surgery on immunodeficient RAG-1 deficient mice (Rag-1^−/−^ mice) and RAG-2/common γ-chain cytokine receptor double knockout mice (Rag-2/cγ^−/−^ mice.) We examined miR expression over a 14-day time course as described [14] and as before, used naïve kidneys for each strain for day zero. We then used PCA on the resulting microarray data. This revealed that the first 10 PCs accounted for 92% of the variance, while the first three PCs account for 65% of variance. Thus, the majority of variance can be analyzed in three dimensions.

**Figure 3:**
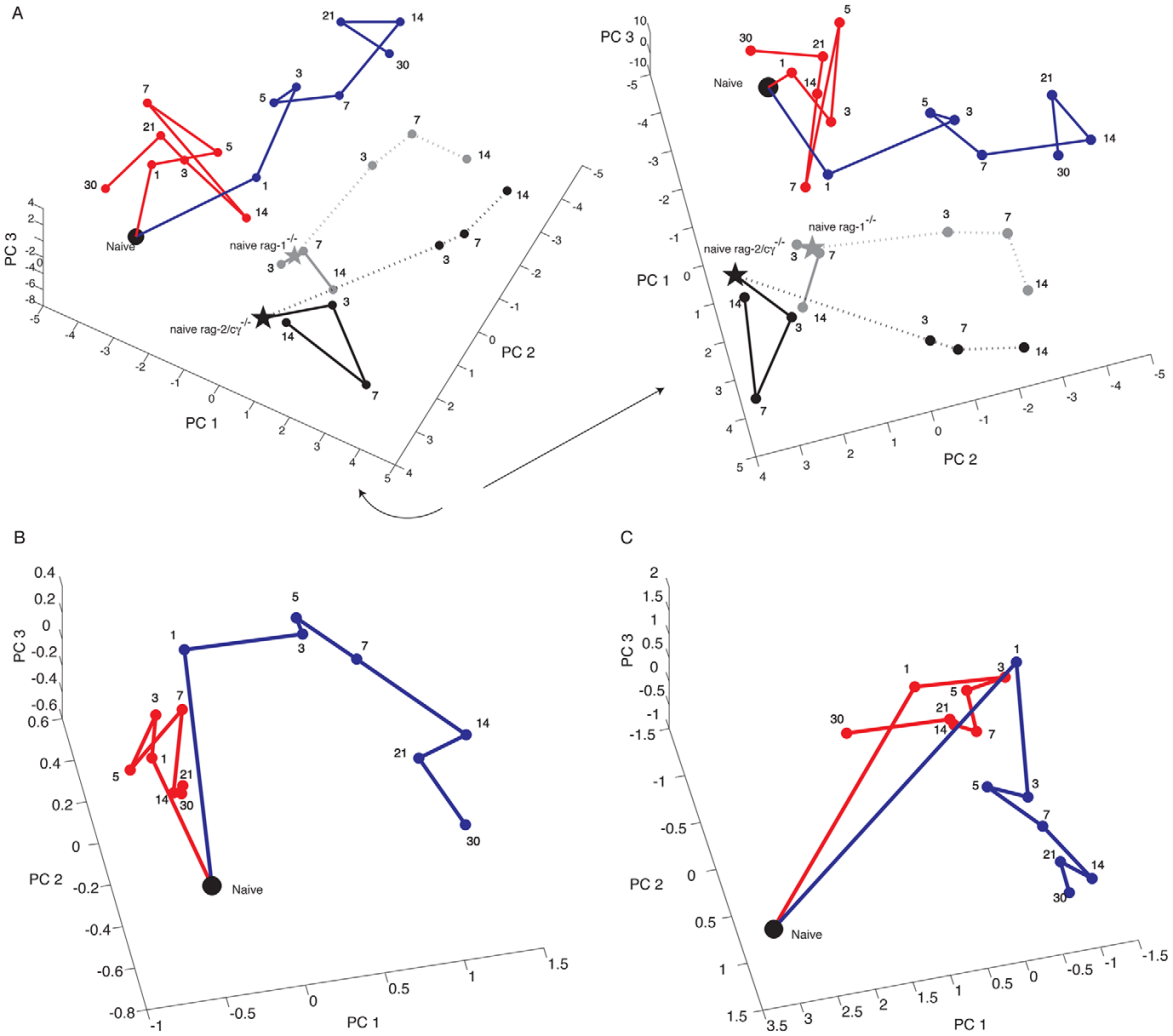
Distinct patterns of miR expression based on PCA are lymphocyte independent and can be detected using a limited set of miRs. Panel A, three-dimensional plot of the first three PCs for C57BL/6 mice undergoing either a sham procedure (red line), or IRI (blue line), Rag-1^−/−^ mice undergoing either a sham procedure (solid grey line) or IRI (dotted grey line), or Rag-2/γc^−/−^ mice undergoing either a sham procedure (solid black line) or IRI (dotted black line). Samples for naïve C57BL/6 mice are show as a black dot. Samples for naïve Rag-1^−/−^ and or Rag-2/cγ^−/−^ mice are shown as grey and black stars, respectively. Right panel, shown is the plot to the left rotated clockwise in order to highlight differences in PC1 between samples (Movie S2). Panel B, PCA of nine differentially expressed miRs. Shown is a three-dimensional plot of the first three PCs obtained by performing PCA on expression data for miR-21, miR-20a, miR-146a, miR-199a-3p, miR-214, miR-192, miR-187, miR-805 and miR-194 obtained for kidneys from C57BL/6 mice following IRI (blue line) or sham surgery (red lines) (Movie S3). Panel C, Shown is a three-dimensional plot of the first three PCs obtained by performing PCA on all expression data obtained for kidneys from C57BL/6 mice following IRI (blue line) or sham surgery (red lines) in which we eliminated miR-21, miR-20a, miR-146a, miR-199a-3p, miR-214, miR-192, miR-187, miR-805 and miR-194 from the analysis. Samples for naïve C57BL/6 mice are show as a black dot with a red center. Numbers shown represent the time point analyzed in days. (Movie S4).

Three-dimensional variance plots of the first three PCs indicated that naive Rag-2/cγ^−/−^ and Rag-1^−/−^ mice exhibit an initial miR expression pattern that is distinct from wild-type C57BL/6 mice prior to any treatment (Fig. 3A). These data suggest that baseline levels of miR expression are different in the kidneys of immunodeficient and immunocompetent mice. In the immunodeficient mice, sham injury resulted in a trajectory that, after day 1, seems to fluctuate around the initial response (Fig. 3A). With respect to this observation they are similar to the C57BL/6 mice. IRI samples from Rag-2/cγ^−/−^ and Rag-1^−/−^ mice also exhibited alterations in miR expression that paralleled those observed for C57BL/6 IRI samples (Fig. 3A). Thus, changes observed in miR expression following IRI in C57BL/6, Rag-2/cγ^−/−^ and Rag-1^−/−^ mice are similar, and distinct from the changes observed in sham controls. These data suggest that the observed alterations in miR expression occur in a lymphocyte independent manner, and most likely reflect a kidney intrinsic signature of renal injury that may be useful as a biomarker.

### Defining the significance of a lymphocyte independent pattern of changes in miR expression

The question arises, are the responses of the immunodeficient mice to IRI similar to those of the C57BL/6 mice? To study this, we performed PCA on the combined data for all groups (Fig. 3A). As before we defined directions for the C57BL/6 IRI mice by taking the day 1 data point and projecting through the subsequent data points. For the immunodeficient mice, we used the day zero as our center of projection. We made this choice because the immunodeficient mice do not show an initial sham response. We then graphed these directions on a common sphere, and measured the angles between them (using spherical standard deviations as we did above to measure separation between the C57BL/6 sham and IRI groups). The Rag-1^−/−^ and C57BL/6 IRI groups are separated by 34.5°, while the Rag-2/cγ^−/−^ and C57BL/6 IRI groups are separated by 43.1°. Under the null hypothesis that the immunodeficient response is random, this degree of proximity would be expected with a *P*-value of 0.02.

### Defining a subset of miRs that can distinguish injured kidneys that underwent IRI

Our previous work showed that in C57BL/6 mice, expression of miR-21, miR-20a, miR-146a, miR-199a-3p, miR-214, miR-192, miR-187, miR-805 and miR-194 is significantly different between IRI and sham control groups at all times analyzed [14]. PCA confirmed that the expression profiles observed for these nine miRs in IRI and sham samples are distinct (Fig. 3B). Sham controls exhibit variation that fluctuated around the day 1 values, reminiscent of what we observed for the entire miR data set (Fig. 1B–C). And from day 1 onward, IRI samples exhibit variation that is visually distinct from sham samples. However, using the first three PCs of variance in these nine miRs, neither the sham nor IRI directions rose to statistical significance.

We also performed PCA on miR expression data in which these nine miRs were eliminated from the full data set, a “digital knockout”. This changed the profile of the 3D-plot that was generated in that the discrimination observed between samples in each groups was reduced (Fig. 3C). However, the direction of variance for samples from sham and IRI treated mice remained significantly different. These data therefore suggest that these nine differentially regulated miRs are not the only miRs in this set that could serve as effective biomarkers of renal IRI, although their expression is different in IRI and sham samples.

## Discussion

Using PCA to examine miR expression over a time course we were able to generate 3 dimensional plots in which it is apparent that over the course of injury, miR expression changes in a distinct fashion that can be described based on the trajectory of the lines obtained by plotting variance for the first 3 PCs. Directions are naturally represented as points on a sphere. Using this property we were able to combine Monte Carlo methods and spherical geometry to assess the statistical significance of these directions. Using this novel approach we were able to determine that observed directions are not random events. We suggest that the apparent directionality of sham and IRI data reflects predictable alterations in miR expression throughout the course of an injury response. Importantly, because this approach allows us to analyze data for individual time points, these results also indicate that the pattern of changes in miR expression following injury is observed as early as day 3 and continues throughout the course of the experiment. This suggests that the overall changes in miR expression we observed are due to a single process of response to injury. Thus, these methods allowed us to determine that miR expression profiling can be used to distinguish between kidneys that have undergone IRI and sham controls and miR expression may therefore be useful as a biomarker for IRI.

Based on PCA miR expression profiles in naïve Rag-2/cγ^−/−^, and Rag-1^−/−^ kidneys do not appear to be the same as observed for naïve C57BL/6 kidneys in that the initial condition described for naive immunodeficient mice results in a plot is distinct from that observed for C57BL/6 even though all mice are on the C57BL/6 background. These data suggest that baseline levels of miR expression are different in the kidneys of immunodeficient and immunocompetent mice. We suggest therefore that it is critical to use caution when comparing the effects of kidney injury in immunodeficient and immunocompetent mice in terms of concluding that differences in results obtained are attributable to immunodeficiency alone since the baseline state of the kidneys from these animals varies dramatically. Nevertheless, our data suggest that the trajectories of miR expression changes following IRI are similar in C57BL/6 and immunodeficient and therefore lymphocyte independent.

We previously suggested that of the miRs analyzed, nine (miR-21, miR-20a, miR-146a, miR-199a-3p, miR-214, miR-192, miR-187, miR-805 and miR-194) stand out as being differentially expressed in C57BL/6 mice undergoing IRI compared to the expression observed in mice undergoing a sham procedure [14]. Expression of these miRs in kidneys from mice that underwent IRI was statistically different from sham kidneys at every time point analyzed. PCA of these nine miR revealed that the expression profiles observed in samples from C57BL/6 mice undergoing IRI and sham controls are distinct. While these nine miRs do not describe a direction for sham samples, it is apparent that these miRs describe changes in IRI samples that are visually similar to those observed when all the miRs are analyzed. Interestingly, these nine miR do not describe a single direction as observed in the complete data set, but appear to vary in a single direction from day 1 through day 14, and then in a similar but separate direction thereafter. We suggest that this may reflect an initial response to injury that is then not maintained long-term. This is suggestive of a model in which a few biologically important miR may be responding in a protective fashion that is independent of the overall changes in miR expression which may have a single direction related to injury and death of the kidney. This also demonstrates that PCA in conjunction with statistical analysis of the direction of variance is a powerful tool to discern patterns of responses following injury.

Digital knockout provided a method to assess the contribution of any given set of miRs to the overall patterns that we have observed. In our digital knockout experiments the differences between the direction of miR expression changes in IRI and sham control groups remained significant. We point out that even though variance was clearly reduced, the differences between the direction of miR expression changes in IRI and sham control groups remained significant. This suggests that the distinction between sham and IRI groups can be detected by assessing expression of many different miRs underscoring the validity of using miR expression as a biomarker of IRI. While we have not yet determined which subset of miRs distinguish IRI and sham samples best, we suggest that this approach can be used to define such a data set, develop novel diagnostic tools and determine which miRs regulate biological processes related to IRI.

The analysis conducted here leads us to conclude that differential expression of miRs might serve as a biomarker of renal injury. However, in order for miR expression to serve as a biomarker it will be important to determine whether miR expression has prognostic value and whether miR expression returns to baseline following a healing response. In terms of translation into a clinical tool, it will be critical to examine whether differential expression of miRs can be observed in the blood or perhaps urine of subjects undergoing IRI. We believe that the data presented here support that notion that such endeavors are worthwhile. We also wish to point out that differential expression of miRs in the context of renal injury also has to potential to reveal the existence of molecular pathways that may be involved in the injury or repair response that can be manipulated to prevent damage or promote healing. Indeed, this type of analysis may reveal novel druggable targets.

## Methods

### Animals

10–12 week old male C57BL/6J, B6.129S7-*Rag1^tm1Mom^*/J (Jackson) and (C57BL/6J×C57BL/10SgSnAi)-[KO]*γc*-[KO]*Rag*2 (Taconic) mice were housed and handled in accordance with institutional policies and procedures. All animal experiments were approved by the Harvard Standing Committee on Animal Use, Protocol #04077.

### IRI and miR Microarray Analysis

Unilateral warm IRI was induced and miR expression analyzed by microarray as in [14]. All data is MIAME compliant and has been deposited in the Geo database. Accession numbers GSE29495.

### Mathematical analysis

#### Normalization

Our data consist of 29 time points organized into 6 time series. Each of these data point consists of expression levels for the 571 miRs of mmu-miRBase 10.0. We thus have
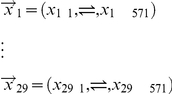
Expression levels below 200 were deemed to be below reliable detection. We thus took 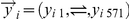, where 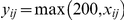. We then normalized expression of each miR to its expression in naïve C57BL/6 mice. Taking 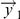 to be expression in naïve mice, we have 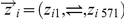 where 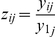. Finally, we took the natural log of normalized expression giving 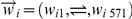, where 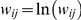. Notice that if a miR never reaches the cutoff level of 200, it appears with the value 0 in each 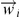.

#### Digital Knock-out

Digital knock-out of a set *S* of miRs was performed by setting 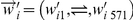 where 
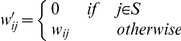
 prior to performing PCA.

#### Computations on the sphere

Given two points *A* and *B*, the displacement from *A* to *B* is their difference *B-A*. The direction from *A* to *B* is 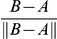, where the vertical bars denote the Euclidean norm. When the points in question are in 3-dimensional space, **R** ^3^, this direction is a point on the unit 2-sphere, **S** ^2^. Given two points 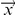 and 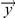 on the 2-sphere, their distance on the sphere is the angle between them, that is, 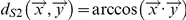. We report these angles in degrees. Given a point 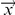 on the sphere, the disk of radius *r* around that point on the sphere consists of all points of the sphere within (spherical) distance *r* of 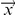.

We have considered time-series, e.g., days 1, 3, 5, 7, 14, 21, 30 of the warm ischemia treated C57 BL/6 mice or days 0, 1, 3, 5, 7, 14, 21, 30 of the sham treated C57BL/6 mice. Let 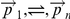 be the projection of such a series onto the first three PCs. This gives *n-1* directions
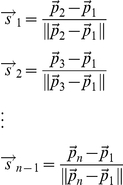
We take 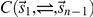 and 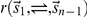 to be the center and the radius of the smallest circle on the unit sphere which contains 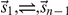. 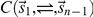 is not necessarily well defined. For example it can be taken to be either the north pole or the south pole if 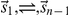 all lie on the equator. However, this happens with probability 0. This computation is closely related to finding the spherical standard deviation of a set of points on the sphere. (See below.)

We used 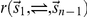 to assess the hypothesis that 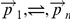 define a specific direction. We take as our null hypothesis, the assumption that this path is a random walk with the displacements 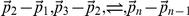 drawn from the uniform distribution on the 2-sphere. We take as the *P*-value for the directionality of 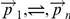, the probability that a random walk of this form produces directions enclosed by a circle whose radius is no greater than 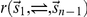.

We used Monte Carlo methods to assess this probability. We generate 10,000 such random walks. This required us to generate points on the 2-sphere randomly chosen from the uniform distribution. This can be done by choosing *x*, *y* and *z* according to the normal distribution with 0 mean and standard deviation 1 and dividing (*x,y,z*) by its norm.

Having generated a random walk 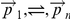, we needed to find the center and radius of the smallest circle containing the directions 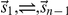. To do this, we performed constrained minimization using Matlab's fmincon function, that is, we minimized maximum distance to 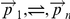 given that the center is constrained to lie on the 2-sphere. Functions such as fmincon are only guaranteed to find local minima. The local minimum found may depend on the initial value chosen as a candidate center and need not be the global minimum. Accordingly, for each set of directions, 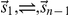, we performed this minimization starting at each of the 8 points 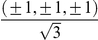, thus giving an initial point in each octant. To test the reliability of this choice, we generated 1000 sets of points on the sphere and probed the efficacy of using these 8 points by generating 20 random starting points for each set. In each case, the 8-point method provided the minimum value to within the tolerance of fmincon. Perhaps surprisingly, starting at the (normalized) mean of 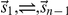, did not always converge to the minimum radius. We generated 10000 random walks and computed the minimum radius circle for each of these. We used the resulting distribution of radii to compute *P*-values.

Given a set of points 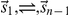, there is a closely related way for finding a center 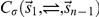 and radius 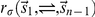. Rather than finding the center which minimizes the angular radius of the smallest enclosing circle, we find the center which minimizes the root mean square of the angular distances to the points 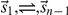. The resulting radius is the spherical standard deviation of these points. The enclosing circle has the advantage of being visually clear. Spherical standard deviation has the advantage of being more robust with respect to outliers. We have used both here. (For further discussion of spherical standard deviation, see [15].)

Finally we note that we have described these computations in the context of 3-dimensional data resulting from the first 3 PCs of our time series, in which case the computations are carried out on the 2-sphere, S^2^. The same sorts of computations can be performed directly on our 144 dimensional data set in which case they are carried out on the 143-sphere, S^143^.

## Supporting Information

Movie S1 **Principal component analysis of miR expression following unilateral warm IRI.** Movie showing rotation of three-dimensional plot of the first three PCs for mice undergoing either a sham procedure (red line), or IRI (blue line). Samples for naïve C57BL/6 mice are shown as a black dot. Numbers shown represent the time point analyzed in days.
(MOV)

Movie S2 **Distinct patterns of miR expression based on PCA are lymphocyte independent.** Movie showing rotation of three-dimensional plot of the first three PCs for C57BL/6 mice undergoing either a sham procedure (red line), or IRI (blue line), Rag-1^−/−^ mice undergoing either a sham procedure (solid grey line) or IRI (dotted grey line), or Rag-2/γc^−/−^ mice undergoing either a sham procedure (solid black line) or IRI (dotted black line). Samples for naïve C57BL/6 mice are show as a black dot. Samples for naïve Rag-1^−/−^ and or Rag-2/cγ^−/−^ mice are shown as grey and black stars, respectively.
(MOV)

Movie S3 **PCA of nine differentially expressed miRs.** Shown is a three-dimensional plot of the first three PCs obtained by performing PCA on expression data for miR-21, miR-20a, miR-146a, miR-199a-3p, miR-214, miR-192, miR-187, miR-805 and miR-194 obtained for kidneys from C57BL/6 mice following IRI (blue line) or sham surgery (red lines).
(MOV)

Movie S4 **PCA of miR expression data without the nine differentially expressed miRs.**Movie showing the rotation of a three-dimensional plot of the first three PCs obtained by performing PCA on all expression data obtained for kidneys from C57BL/6 mice following IRI (blue line) or sham surgery (red lines) in which we eliminated miR-21, miR-20a, miR-146a, miR-199a-3p, miR-214, miR-192, miR-187, miR-805 and miR-194 from the analysis. Samples for naïve C57BL/6 mice are show as a black dot with a red center. Numbers shown represent the time point analyzed in days.
(MOV)
